# Supplementary material for: Epitope Mapping of the Diphtheria Toxin and Development of an ELISA-Specific Diagnostic Assay
Source: Vaccines (Basel). 2021 Mar 26;9(4):313. doi: 10.3390/vaccines9040313 (PMC8066203; doi:10.3390/vaccines9040313)
Supplement: Supplementary file 1 [file vaccines-09-00313-s001.pdf]

Table S1: List of synthesized peptides covering the entire sequence of DTx. (F3,F4,F5, F11,F12,F13 positive controls and F9,F17 negative controls.

Spot	Sequence
A1	GSGSGMSRKLFASIL
A2	MSRKLFASILIGALL
A3	FASILIGALLGIGAP
A4	IGALLGIGAPPSAHA
A5	GIGAPPSAHAGADDV
A6	PSAHAGADDVVDSSK
A7	GADDVVDSSKSFVME
A8	VDSSKSFVMENFSSY
A9	SFVMENFSSYHGTKP
A10	NFSSYHGTKPGYVDS
A11	HGTKPGYVDSIQKGI
A12	GYVDSIQKGIQKPKS
A13	IQKGIQKPKSGTQGN
A14	QKPKSGTQGNYDDDW
A15	GTQGNYDDDWKGFYS
A16	YDDDWKGFYSTDNKY
A17	KGFYSTDNKYDAAGY
A18	TDNKYDAAGYSVDNE
A19	DAAGYSVDNENPLSG
A20	SVDNENPLSGKAGGV
A21	NPLSGKAGGVVKVTY
A22	KAGGVVKVTYPGLTK
A23	VKVTYPGLTKVLALK
A24	PGLTKVLALKVDNAE
D1	QSIALSSLMVAQAIP
D2	SSLMVAQAIPLVGEL
D3	AQAIPLVGELVDIGF
D4	LVGELVDIGFAAYNF
D5	VDIGFAAYNFVESII
D6	AAYNFVESIINLFQV
D7	VESIINLFQVVHNSY
D8	NLFQVVHNSYNRPAY
D9	VHNSYNRPAYSPGHK
D10	NRPAYSPGHKTQPFL
D11	SPGHKTQPFLHDGYA
D12	TQPFLHDGYAVSWNT
D13	HDGYAVSWNTVEDSI
D14	VSWNTVEDSIIRTGF
D15	VEDSIIRTGFQGESG
D16	IRTGFQGESGHDIKI
D17	QGESGHDIKITAENT
D18	HDIKITAENTPLPIA
D19	TAENTPLPIAGVLLP
D20	PLPIAGVLLPTIPGK
D21	GVLLPTIPGKLDVNK
D22	TIPGKLDVNKSKTHI
D23	LDVNKSKTHISVNGR
D24	SKTHISVNGRKIRMR

Spot	Sequence
B1	VLALKVDNAETIKKE
B2	VDNAETIKKELGLSL
B3	TIKKELGLSLTEPLM
B4	LGLSLTEPLMEQVGT
B5	TEPLMEQVGTEEFIK
B6	EQVGTEEFIKRFGDG
B7	EEFIKRFGDGASRVV
B8	RFGDGASRVVLSLPF
B9	ASRVVLSLPFAEGSS
B10	LSLPFAEGSSSVEYI
B11	AEGSSSVEYINNWEQ
B12	SVEYINNWEQAKALS
B13	NNWEQAKALSVELEI
B14	AKALSVELEINFETR
B15	VELEINFETRGKRGQ
B16	NFETRGKRGQDAMYE
B17	GKRGQDAMYEYMAQA
B18	DAMYEYMAQACAGNR
B19	YMAQACAGNRVRRSV
B20	CAGNRVRRSVGSSLS
B21	VRRSVGSSLSCINLD
B22	GSSLSCINLDWDVIR
B23	CINLDWDVIRDKTKT
B24	WDVIRDKTKTKIESL
E1	SVNGRKIRMRCRAID
E2	KIRMRCRAIDGDVTF
E3	CRAIDGDVTFCRPKS
E4	GDVTFCRPKSPVYVG
E5	CRPKSPVYVGNGVHA
E6	PVYVGNGVHANLHVA
E7	NGVHANLHVAFHRSS
E8	NLHVAFHRSSSEKIH
E9	FHRSSSEKIHSNEIS
E10	SEKIHSNEISSDSIG
E11	SNEISSDSIGVLGYQ
E12	SDSIGVLGYQKTVDH
E13	VLGYQKTVDHTKVNS
E14	KTVDHTKVNSKLSLF
E15	TKVNSKLSLFFEIKS
E16	KLSLFFEIKSGSGSG
E17	
E18	
E19	
E20	
E21	
E22	
E23	
E24	

Spot	Sequence
C1	DKTKTKIESLKEHGP
C2	KIESLKEHGPIKNKM
C3	KEHGPIKNKMSESPN
C4	IKNKMSESPNKTVSE
C5	SESPNKTVSEEKAKQ
C6	KTVSEEKAKQYLEEF
C7	EKAKQYLEEFHQTAL
C8	YLEEFHQTALEHPEL
C9	HQTALEHPELSELKT
C10	EHPELSELKTVTGTN
C11	SELKTVTGTNPVFAG
C12	VTGTNPVFAGANYAA
C13	PVFAGANYAAWAVNV
C14	ANYAAWAVNVAQVID
C15	WAVNVAQVIDSETAD
C16	AQVIDSETADNLEKT
C17	SETADNLEKTTAALS
C18	NLEKTTAALSILPGI
C19	TAALSILPGIGSVMG
C20	ILPGIGSVMGIADGA
C21	GSVMGIADGAVHHNT
C22	IADGAVHHNTEEIVA
C23	VHHNTEEIVAQSIAL
C24	EEIVAQSIALSSLMV
F1	
F2	
F3	KEVPALTAVETGATN
F4	
F5	GYPKDGNAFNNLDRI
F6	
F7	YDYDVPDYAGYPYDV
F8	
F9	QEVRKYFCV
F10	
F11	KEVPALTAVETGATN
F12	
F13	GYPKDGNAFNNLDRI
F14	
F15	YDYDVPDYAGYPYDV
F16	
F17	QEVRKYFCV
F18	
F19	
F20	
F21	
F22	
F23	
F24	

Figure 1. List of the synthesized peptides of DTX covering the sequence aa518 to aa547, and probed with miVS (A), huVS (B), and hoThe sera (C). In black, the sequences considered reactive to antibodies are marked, using a 50% cut-off. The membrane is shown in figure 2.

	A	B	C
A1	RSSSEKIHSNEISSD	RSSSEKIHSNEISSD	RSSSEKIHSNEISSD
A2	SSSEKIHSNEISSDS	SSSEKIHSNEISSDS	SSSEKIHSNEISSDS
A3	SSEKIHSNEISSDSI	SSEKIHSNEISSDSI	SSEKIHSNEISSDSI
A4	SEKIHSNEISSDSIG	SEKIHSNEISSDSIG	SEKIHSNEISSDSIG
A5	EKIHSNEISSDSIGV	EKIHSNEISSDSIGV	EKIHSNEISSDSIGV
A6	KIHSNEISSDSIGVL	KIHSNEISSDSIGVL	KIHSNEISSDSIGVL
A7	IHSNEISSDSIGVLG	IHSNEISSDSIGVLG	IHSNEISSDSIGVLG
A8	HSNEISSDSIGVLGY	HSNEISSDSIGVLGY	HSNEISSDSIGVLGY
A9	SNEISSDSIGVLGYQ	SNEISSDSIGVLGYQ	SNEISSDSIGVLGYQ
A10	NEISSDSIGVLGYQK	NEISSDSIGVLGYQK	NEISSDSIGVLGYQK
A11	EISSDSIGVLGYQKT	EISSDSIGVLGYQKT	EISSDSIGVLGYQKT
A12	ISSDSIGVLGYQKTV	ISSDSIGVLGYQKTV	ISSDSIGVLGYQKTV
A13	SSDSIGVLGYQKTVD	SSDSIGVLGYQKTVD	SSDSIGVLGYQKTVD
A14	SDSIGVLGYQKTVDH	SDSIGVLGYQKTVDH	SDSIGVLGYQKTVDH
A15	DSIGVLGYQKTVDHT	DSIGVLGYQKTVDHT	DSIGVLGYQKTVDHT

Figure S2: Alignment of the primary structure of DTx [(1-*C. diphtheria* (Q6NK15), 2-*C. ulcerans* (AQA1Y0 HBB0) and 3-*C. pseudotuberculosis* (WP_014654963.1)] showing in grey the epitopes (CB/DTx1-20) identified by Spot-synthesis.

```
          10         20         30         40         50         60         70         80
1-MSRKLFASIL IGALLGIGAP PSAHAGADDV VDSSKSFVME NFSSYHGTKP GYVDSIQKGI QKPKSGTQGN YDDDWKGFYS
2-MNRKLFASIL IGALLGIGTP LSAHASADDV VDSSKSFVME NFSSYHGTKP GYVDSIQKGI QKPKSGTQGN YDDDWKGFYS
3-MNRKLFALIL IGALLGIGAP LSAHASVDDV VDSSKSFVME NFSSYHGTKP GYVDSIQKGI QKPKSGTQGN YDDDWKGFYS
  ---------------------CB/Tx-1------- ---CB/Tx-2------------CB/Tx-3-------------------------------CB/Tx-4

          90        100        110        120        130        140        150        160
1-TDNKYDAAGY SVDNENPLSG KAGGVVKVTY PGLTKVLALK VDNAETIKKE LGLSLTEPLM EQVGTEEFIK RFGDGASRVV
2-TDNKYDAAGY SVDNENPLSG KAGGVVKVTY PGLTKILALK VDNAETIKKE LGLSLTEPLM EQVGSEEFIK RFGDGASRVV
3-TDNKYDAAGY SVDNENPLSG KAGGVVKVTY PGLTKILALK VDNAETIKKE LGLSLTEPLM EQVGSEEFIK RFGDGASRVV
  ----------------CB/TX-5-------------------------------------------CB/Tx-6----------------------CB/TX----

         170        180        190        200        210        220        230        240
1-LSLPFAEGSS SVEYINNWEQ AKALSVELEI NFETRGKRGQ DAMYEYMAQA CAGNRVRRSV GSSLSCINLD WDVIRDKTKT
2-LSLPFAEGSS SVEYINNWEQ AKALSVELEI NFETRGKRGQ DAMYEYMAQS CAGNRVRRSV GNSSSCINLD WDAIRDKTKT
3-LSLPFAEGSS SVEYINNWEQ AKALSVELEI NFENRGKRGQ DAMYEYMAQS CAGNRIRRSV SNSSSCLNLD WDAIRDKTKA
  ----CB/Tx-8------------------------------------CB/Tx-9-------------------------------------------------

         250        260        270        280        290        300        310        320
1-KIESLKEHGP IKNKMSESPN KTVSEEKAKQ YLEEFHQTAL EHPELSELKT VTGTNPVFAG ANYAAWAVNV AQVIDSETAD
2-KIESLKEHGP IKNKMSESPN KAVSEEKAKQ YLEEFHQTAL EHPELSELKT VTGTNSVFAG ANYAAWAVNV AQVIDSETAD
3-KIESLKENGP IKNKMSESPN KTVSEEKAKQ YLEEFHQTAL EHPELSELKT VAETNSVFAG ANYAAWAVNV AQVIDSKTAD
  -------------CB/Tx-10------------------------CB/Tx-11-----------------------------CB/Tx-12--------CB/Tx-13

         330        340        350        360        370        380        390        400
1-NLEKTTAALS ILPGIGSVMG IADGAVHHNI EEIVAQSIAL SSLMVAQAIP LVGELVDIGF AAYNFVESII NLFQVVHNSY
2-NLEKTTAALS ILPGIGSVMG IADGAVHHNT EEIVAQSIAL SSLMVAQAIP LVGELVDIGF AAYNFVESII NLFQVVHNSY
3-NLEKTTAALS ILPGIGSVMG IADGAVHHNT EEIVAQSIAL SSLMIAQAIP LVGELVDIGF AAYNFVESII NLFQVIHNSY
  ------------------------------------CB/Tx-14----------------------------------CB/Tx-15--------------------------

         410        420        430        440        450        460        470        480
1-NRPAYSPGHK TQPFLHDGYA VSWNTVEDSI IRTGFQGESG HDIKITAENT PLPIAGVLLP TIPGKLDVNK SKTHISVNGR
2-NRPAYSPGHK TQPFLHDGYA VSWNTVEDSI IQTGFQGESG HDIKITAENT PLPIAGVLLP TIPGKLDVNK SKTHISVNGR
3-NRPTYSPGHK TQPFLHDGYA VSWNTVEDSI IKTGFQGESG HDIKITAENT PLPIAGVLLP TITGKLDVNK SKTHISVNGR
  --------------CB/Tx-16----------------------------CB/Tx-17-----------------------------------------CB/Tx-18

         490        500        510        520        530        540        550        560
1-KIRMRCRAID GDVTFCRPKS PVYVGNGVHA NLHVAFHRSS SEKIHSNEIS SDSIGVLGYQ KTVDHTKVNS KLSLFFEIKS
2-KIRMRCRAID GDVTFCRPKS PVYVGNGVHA NLHVAFHRSS SEKIHSNEIS SDSIGVLGYQ KIVDHTKVNS KLSLFFEIKS
3-KIRMQCRAID GNVTFCRPKS PVYVGKGVHA NLHVAFHRSN SEKIQSNEIL SDSIGILGYQ KIVDHTKVNS KLSLFFEIKS
  ---------------CB/Tx-19------------------------------------------CB/Tx-20---------------------------------
```
